# Early 2-Factor Transcription Factors Associated with Progression and Recurrence in Bevacizumab-Responsive Subtypes of Glioblastoma

**DOI:** 10.3390/cancers16142536

**Published:** 2024-07-14

**Authors:** Jian Shi

**Affiliations:** Department of Neurology, San Francisco Veterans Affairs Health Care System and University of California, San Francisco, CA 94121, USA; jian.shi@ucsf.edu

**Keywords:** glioblastoma, E2F family, Bevacizumab, recurrence, machine learning algorithm, bioinformatics

## Abstract

**Simple Summary:**

This study has provided new insights into the role of E2F transcription factors, a group of proteins, in glioblastoma (GBM), a type of brain cancer. These proteins play a part in various processes that can lead to cell death, growth, and the formation of new blood vessels, all of which are crucial in the development of GBM. To understand how these proteins impact a specific type of GBM that responds to the drug Bevacizumab (BVZ), researchers utilized advanced computer algorithms on datasets from multiple large databases. By doing so, they were able to predict the potential lifespan of GBM patients based on the levels of these proteins found in their tumors. The study revealed that an increase in a protein called E2F8 and its regulatory networks following BVZ treatment might be associated with the recurrence of cancer. This suggests that E2F8 could be a key factor in the reemergence of GBM after treatment. Furthermore, the study indicated that BVZ could be detrimental to GBM patients whose tumors do not respond to the drug, potentially worsening the disease. These findings emphasize the significance of E2F proteins in the aggressiveness of GBM and its response to BVZ, suggesting their potential as markers for predicting patient outcomes and as targets for more personalized treatment approaches.

**Abstract:**

The early 2-factor (E2F) family of transcription factors, including E2F1 through 8, plays a critical role in apoptosis, metabolism, proliferation, and angiogenesis within glioblastoma (GBM). However, the specific functions of E2F transcription factors (E2Fs) and their impact on the malignancy of Bevacizumab (BVZ)-responsive GBM subtypes remain unclear. This study used data from The Cancer Genome Atlas (TCGA), Chinese Glioma Genome Atlas (CGGA), European Molecular Biology Laboratory’s European Bioinformatics Institute (EMBL-EBI), and Gene Expression Omnibus (GEO) to explore the impact of eight E2F family members on the clinical characteristics of BVZ-responsive GBM subtypes and possible mechanisms of recurrence after BVZ treatment. Using machine learning algorithms, including TreeBagger and deep neural networks, we systematically predicted and validated GBM patient survival terms based on the expression profiles of E2Fs across BVZ-responsive GBM subtypes. Our bioinformatics analyses suggested that a significant increase in E2F8 post-BVZ treatment may enhance the function of angiogenesis and stem cell proliferation, implicating this factor as a candidate mechanism of GBM recurrence after treatment. In addition, BVZ treatment in unresponsive GBM patients may potentially worsen disease progression. These insights underscore that E2F family members play important roles in GBM malignancy and BVZ treatment response, highlighting their potential as prognostic biomarkers, therapeutic targets, and recommending precision BVZ treatment to individual GBM patients.

## 1. Introduction

Glioblastoma (GBM) is a highly malignant tumor that primarily affects the central nervous system and is the most common primary intracranial tumor. The fifth edition of the World Health Organization (WHO) classification of tumors of the central nervous system delineates GBM as a distinct subtype, different from the previously described glioblastoma multiforme [1]. This GBM subtype is specifically restricted to adult grade 4 diffuse astrocytic gliomas that are *wild type* for isocitrate dehydrogenase (IDH). In fact, mutations in IDH and methylation of the O6-methylguanine-DNA methyltransferase (MGMT) gene in GBM are associated with a more favorable prognosis. Historically, GBM treatment has involved a combination of surgical resection, radiation therapy, and chemotherapy. Despite the high likelihood of tumor recurrence and progression, the integration of multimodal treatment approaches has led to gradual improvement in patient outcomes. Median overall survival has been extended to approximately 15 months, and progression-free survival has improved to 10 months. The five-year survival rate stands at 6.8% [2,3,4]. These advances underscore the importance of continued research and the necessity for ongoing innovation in the management of GBM.

Navigating the complex world of glioblastoma (GBM) chemotherapies [2,5], these studies provide a comprehensive overview of current therapeutic practices, emerging strategies, and the challenges within this therapeutic domain. Temozolomide (TMZ), an oral DNA alkylating agent that penetrates the blood–brain barrier, is a first-line systemic treatment for glioblastoma, usually in combination with radiation therapy. However, the effectiveness of TMZ depends on the methylation status of the MGMT gene promoter. Patients with methylated genes tend to have better treatment outcomes because their tumor cells have reduced DNA repair capacity. Despite initial responses, resistance and relapse are common, leading to consideration of alternative chemotherapeutic agents such as other alkylating agents. Antiangiogenic approaches, including bevacizumab (BVZ), a monoclonal antibody against vascular endothelial growth factor (VEGF), have been shown to prolong progression-free survival in relapsed cases, but their overall survival benefit has been inconsistent. Immunotherapy, including vaccines, oncolytic viruses, immune checkpoint inhibitors, and CAR-T cell therapy, is an exciting frontier in glioblastoma treatment that harnesses the power of the immune system. In fact, their widespread use is limited by several obstacles, including the lack of tumor-specific antigens, ineffective cell trafficking, treatment-related toxicities, and an immunosuppressive tumor microenvironment. The study of novel targets and synthetic agents, natural compounds, and immunotherapy has further expanded the therapeutic prospects of glioblastoma, bringing hope for treating drug resistance and relapse. However, the heterogeneity of glioblastoma, strong blood–brain barrier, constant recurrence, and immunosuppressive tumor microenvironment continue to pose major challenges to the effectiveness of chemotherapy and emerging treatment modalities.

The early 2-factor (E2F) family consists of eight key members in humans: E2F1–8. These members have highly similar DNA-binding domains that directly interact with consensus sequences [6]. The members are categorized into three subgroups based on sequence similarity and functional activity: activator proteins (E2F1-3), atypical repressors (E2F7-8), and canonical repressors (E2F4-6). Each member has distinct expression and functional patterns that align with their subgroup classification [7,8]. E2Fs play a crucial role in the cell cycle by forming the core transcriptional axis. In addition to cell cycle regulation, E2Fs also influence various biological pathways that contribute to malignant progression, such as apoptosis, angiogenesis, and metabolism in several cancers including glioblastoma [9,10,11]. Overexpression of E2F1 in glioma cells has been associated with enhanced proliferation, regulated by miRNAs, including miR-342-3p and miR-377 [12]. Conversely, suppression of E2F1 via siRNA in the GBM U87MG cell line leads to reduced cell proliferation, increased cell death, and diminished neurosphere formation, indicating a role for E2F1 in maintaining GBM stem cells [13]. The silencing of E2F5 has similarly been shown to inhibit the proliferative capacity of GBM cells, while targeting E2F8 is implicated in the regulation of glioma development [14,15]. Furthermore, E2F4 and E2F5 have been demonstrated to directly regulate NF-κB-inducing kinase transcription, playing a crucial role in promoting GBM cell invasion [16]. Notably, E2F1 and E2F2 are associated with increased expression of pro-angiogenic genes in breast cancer, potentially contributing to a more aggressive phenotype [17]. Therefore, E2F members may serve as promising therapeutic targets and potential biomarkers for specific cancers.

Recently, artificial intelligence (AI), encompassing machine learning (ML) and deep learning (DL) methods, has become a powerful tool for classifying cancer subtypes and predicting cancer progression, offering improved accuracy and novel insights over traditional approaches [18,19]. Utilizing AI technologies, such as machine learning and specific deep learning methods, various omics data, including gene expression, RNA sequences, non-coding RNA such as miRNA, and protein expression and modification, can be analyzed to classify cancer subtypes, predict cancer progression, and evaluate treatment response. For example, we have recently used machine learning methods and miRNAs (miR-10b, miR-21, and miR-197) as biomarkers to classify bevacizumab (BVZ) responsive subtypes in glioblastoma (GBM) [20]. Their clinical and pathological characteristics showed that these GBM BVZ responsive patients had significantly shorter overall survival (OS), with a median survival rate of approximately 10 months, compared with 15 months for other GBM patients. The effect of BVZ on the OS may have been masked due to the shorter OS. Furthermore, several genes (ANXA2, HOXD10, EFNA1, HOXD11, ANXA2P2, GREB1L, and FKBP9) were selected as gene markers due to significant changes in expression between these two BVZ response subtypes. In addition, their VEGF methylation levels were significantly lower than those of other GBM patients, whereas their MGMT methylation levels did not affect the OS in BVZ-responsive GBM patients. This study can facilitate precision therapy for GBM patients and further elucidate underlying mechanisms.

Despite responding to treatment, including BVZ therapy, most GBM cases eventually relapse. The underlying mechanisms behind this relapse have been extensively studied but remain unclear. BVZ therapy targets VEGF and inhibits angiogenesis within the tumor. Current opinion suggests that BVZ therapy may prolong progression-free survival (PFS) in GBM treatment, but there is no evidence of its effect on overall survival (OS). After BVZ treatment, some glioblastomas can stimulate the growth of tumor vessels by expressing various angiogenic factors or elevated expression levels, such as EGFR vIII-positive GBM with BVZ treatment [21] and enhanced bFGF (Fibroblast Growth Factor 2) expression [22], showing worse outcomes of BVZ treatment. In addition, research has shown that post-treatment recurrent GBM is also driven by pre-existing, treatment-resistant stem-like cells present in the tumor microenvironment [23]. Moreover, genetic reprogramming and differential oncogene activation, and regulation of self-renewal, tumorigenicity, and metastasis in the relapsed tumor cells and their microenvironments contribute to GBM recurrence [24,25]. By examining these aspects of relapse mechanisms, we can enhance our understanding of GBM development and progression, potentially leading to the discovery of new targets and more effective treatments.

Our previous research identified several key transcription factors, including E2F family members, involved in GBM treated with BVZ [26]. Despite BVZ’s impact on patient prognosis, significant improvements in overall survival (OS) outcomes have not been consistently demonstrated, and tumor recurrence continues to be a major challenge in GBM treatment. Therefore, this study investigates the influence of E2F family members on the malignancy of BVZ-responsive GBM subtypes before and after treatment, integrating the clinicopathological characteristics of the patients with multiple omics data for comprehensive machine learning analyses. It aims to elucidate the molecular mechanisms underlying recurrence and progression following BVZ treatment in GBM patients, and to offer guidance for future therapeutic and translational research strategies.

## 2. Materials and Methods

### 2.1. Data Collection

In this study, mRNA expression profiles, along with clinico-pathological data of glioblastoma (GBM), were obtained from several GBM databases. A total of 426 GBM samples were obtained from the Cancer Genome Atlas (TCGA) data portal (http://cancergenome.nih.gov/dataportal/ accessed on 16 April 2023) [27]. These samples were all from primary tumors, including IDH wild-type and mutant types, as they were determined before the fifth edition of the WHO classification. A total of 136 adult GBM samples were included, comprising primary, recurrent, and secondary tumors from the Chinses Glioma Genome Atlas (CGGA), which have IDH genotype information [28,29]. The dataset GSE7967 of GBM patients treated with Bevacizumab (BVZ) was downloaded from the Gene Expression Omnibus (GEO). Sequencing data from GBM tissues, collected before and after BVZ treatment, were processed using Trimmomatic and subsequently mapped to the human genome (hg19) [30,31]. These gene expressions were quantified as fragments per kilobase million (FPKM). Additionally, a dataset of 15 GBM samples from EMBL-EBI from E-MTAB-1380 and E-MEXP-3296 was included, and the samples were fully paired before and after BVZ treatment [32,33]. For analytical purposes, data from the same patients, pre- and post-BVZ treatment, were paired to facilitate comparative statistical analysis.

The inclusion and exclusion criteria adopted for the datasets used in this study were as follows: patients who received chemotherapy were excluded from the TCGA dataset, whereas patients were included if both miRNA and mRNA expression data were available (426). Patients with no survival time (<5%) were excluded when calculating risk scores and hazard ratios for TCGA and CGGA datasets. In the GSE79671 dataset, there were 7 BVZ responders and 14 BVZ non-responders, but the complete paired patients included 12 BVZ non-responders and 5 BVZ-responsive GBM patients (we obtained). Unlike the paired *t*-test that excluded nonpaired (lacking pre- or post-BVZ treatment data) patients, the conventional *t*-test was performed using the 14 BVZ non-responders and 7 BVZ-responsive patients. In this study, outliers were excluded if (outlier − mean) > 2 × SD.

### 2.2. Bioinformatics Analysis

To explore the potential functions of the eight E2Fs in GBM, several bioinformatics tools were applied to determine the associations between E2Fs expression patterns and the clinical characteristics of samples from the TCGA, CGGA, EMBL-EBI, and GSE79671 datasets. A protein–protein interaction (PPI) network of the eight E2Fs and other differentially expressed (DE) genes was constructed using the STRING database (https://string-db.org/ accessed on 2 May 2024), Cytoscape (3.7.1) software, and Bioinformatics [26,34]. For E2Fs expression in GBM patients and normal individuals, DoSurvive and its specimens were used [35]. Gene Ontology (GO) and Kyoto Encyclopedia of Genes and Genomes (KEGG) pathway analyses were performed by DE genes between pre- and post-BVZ treatment for BVZ responsive subtypes of GBM.

### 2.3. Software and Statistical Methods

In this study, the software and online tools used included R-project (version 4.0.4, www.r-project.org), MatLab2023a (https://www.mathworks.com), Venny 2.1.0 (https://bioinfogp.cnb.csic.es/tools/venny/ accessed on 8 May 2024), MeV (version 4.9.0, MEV, LLC., Walnut Creek, CA, USA, http://www.tm4.org/mev.html accessed on 8 May 2024), g:Profiler (ELIXIR, Tartu, Estonia, https://biit.cs.ut.ee/gprofler/ accessed on 8 May 2024), Bioinformatics (https://bioinformatics.com.cn), and Prism 9.0 (GraphPad Software, Inc. CA, USA). The statistical methods used included Student’s *t*-test, the standard Bonferroni adjusted *t*-test, paired *t*-test, and the Wilcoxon-Mann-Whitney test if the data distribution was not normal.

The risk score model for GBM was constructed using the least absolute shrinkage and selection operator (LASSO) model, based on TCGA data. In this study, the R language package “carnet” was utilized to implement the LASSO multivariate Cox regression model to reduce the number of E2F family members and identify significant members [36]. Following LASSO regression, important members were selected, and their coefficients were utilized to construct the risk score model as follows:

(1) $$Riskscore=\sum_{k=1}^{n}\left(COEFi\times EXPi\right)$$

where COEFi is each of the coefficients, and EXPi is the normalized expression of each selected signature gene.

### 2.4. Variable Selection, TreeBagger, and Deep Neual Network Model Construction

We employed TreeBagger (TB), one of the random forest algorithms, for further prediction and assessment of patient survival after BVZ treatment for GBM patients [37]. The TCGA dataset was classified by miRNA biomarkers and the SVM algorithm, and the GSE79671 dataset was classified by CT scan before and after BVZ treatment for GBM patients. E2F1–8 expression data were extracted from TCGA and GSE79671 and classified into BVZ responsive subtype and the non-responsive subtype of GBM, and the pre- and post-BVZ treatment for the BVZ responsive subtypes of GBM.

Based on the clinical survival data, the survival criteria were classified as:

Criteria = [1, OS ≤ 450 days; 2, OS > 450 days](2)

All parameters in TB were optimized, including tree number, leaf splits, and number to even. One process of selecting tree numbers is shown in the results section. After the TB model had been built, the TCGA dataset was used as the training set and GSE79671 was used as the testing and predicted set. In these processes, Pearson’s correlation was also used to select variables.

For further study, we used a deep neural network (DNN) model to predict patient survival terms defined as the criteria. The E2F1–8 expression data from TCGA and the BVZ responsive subtype categories were combined as input data, so there were 9 input neurons and 2 output neurons. After slight optimization of the linear structure, 60 layers and 16 minibatches were selected in the linear neural network structure. In the process of constructing the DNN model, the E2F8 expression data and BVZ subtype classification in the TCGA dataset were divided into a training set (70%), a test set (15%), and a validation set (15%). The training set was used to train the model, the validation set was used to help fine-tune the model, and the test set was used to provide the model’s prediction accuracy. Furthermore, a CGGA dataset was used to validate the prediction.

## 3. Results

### 3.1. Differential Transcription Factor Expression in Bevacizumab-Responsive Glioblastoma Post-BVZ Treatment

**Figure 1:**
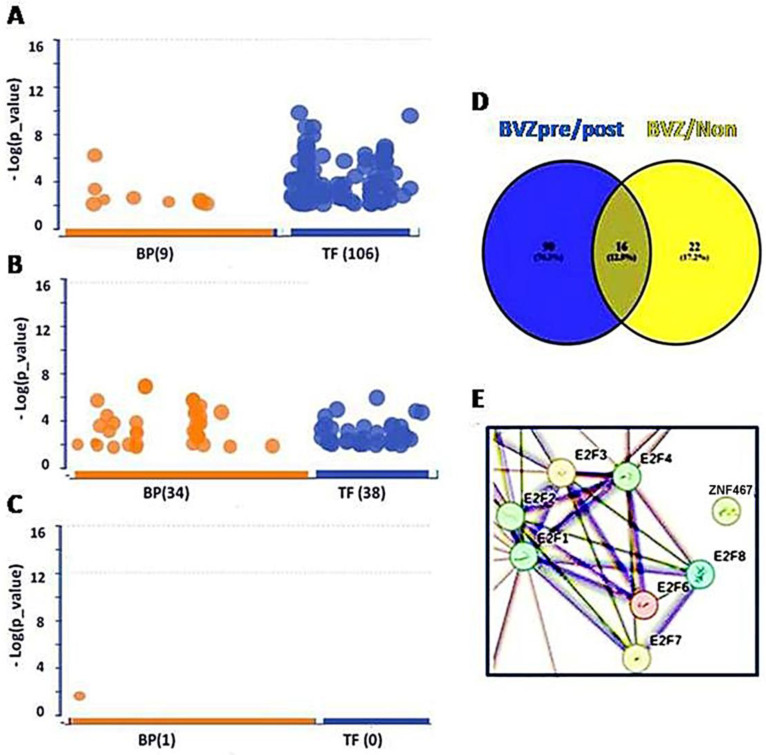
Transcription factors (TFs) involved in BVZ-responsive subtypes of GBM after treatment. (**A**) Image showing the involvement of biological processes (BP (9), orange) of GO and TF (106, blue) between pre- and post-BVZ treatment in responsive GBM using GSE79671 datasets. (**B**) Engagement of BP (34) and TF (38) in GO between BVZ responsive subtypes of GBM based on TCGA datasets. (**C**) Involvement of BP (1) of GO and TF (0) between pre- and post-BVZ treatment in non-responsive subtype of GBM based on GSE79671. In these three analyses, *p* = 0.01 was set as significant. (**D**) Venn diagram showing that 90 TFs involved in pre- and post-BVZ treatment in responsive GBM from the GSE79671 dataset were different from 38 TFs between BVZ responsive subtypes in the untreated TCGA GBM dataset. (**E**) The PPI figure from STRING analysis using 90 TFs shows that E2F members were involved as hub genes.

To explore the impact of bevacizumab (BVZ) treatment on BVZ-responsive glioblastoma (GBM) subtypes, we performed a comprehensive analysis of functional assays including Gene Ontology (GO), Kyoto Encyclopedia of Genes and Genomes (KEGG), and transcription factors (TF). Our analysis involved comparing differentially expressed (DE) genes obtained from 426 TCGA datasets between BVZ-responsive subtypes identified using miRNA biomarkers and machine learning approaches [20], with DE genes being used from 21 datasets (17 samples had full paired data, including before and after treatment data) of GBM patients using a CT scan before and after BVZ treatment to identify BVZ-responsive subtypes [31]. While typical changes were observed in GO and KEGG pathways, striking alterations were detected in TFs expression, particularly evident in Figure 1A.

When analyzing the 557 DE genes from GSE79671, 106 TFs surpassed the significance threshold (set at *p* = 0.01), as illustrated in Figure 1A. In the previous study, 84 TFs passed the significance threshold in BVZ-responsive GBM patients using different parameters, but no TFs passed the threshold in the other two groups [26]. This difference may be attributed to the improvement of our analytical methods and the continuous updating of the data sources we refer to. Therefore, we ensured that the current analysis was performed using the latest and most relevant available data, and we took care to perform these analyses on the same day to maintain consistency. Notably, only 36 TFs met the threshold using the 1000 DE genes from the TCGA dataset (Figure 1B). In contrast, in BVZ-non-responsive patients, no significant TFs were identified between pre- and post-BVZ treatment using the 326 DE genes, not even other terms except one (Figure 1C), indicating non-specific treatment. All three analyses were set at the same parameters for those functional analyses, and we rechecked them on the same day (8 May 2024). Details of significant 106 TFs, including names, sequences, and *p*-values are provided in Supplementary Table S1. These substantial differences underscore the importance of further investigation into TF expression dynamics, especially in the BVZ-responsive subtype of GBM.

Furthermore, we explored the individual differences between 106 TFs from GSE79671 and 38 TFs from TCGA. In Figure 1D, the Venn diagram shows that significant 90 TFs based on GSE79671 pre- and post- BVZ treatment, were not associated with the 38 TFs between BVZ responsive subtypes obtained from TCGA glioblastoma dataset. In Supplementary Table S1, TFs not selected are marked with “#”, showing 90 TFs within 106 TFs. After this, using STRING analysis, the protein–protein interaction (PPI) network of these 90 TFs, including 7 of E2F1–8, (namely E2F1, E2F2, E2F3, E2F4, E2F6, E2F7, and E2F8), was performed and revealed these E2Fs as hub genes, as shown in Figure 1E. This indicates that they play a key role in malignant cells. Supplementary Figure S1 shows the full interaction of these 90 TFs in BVZ-responsive GBM patients treated with BVZ, where the rectangular area is shown in Figure 1E.

### 3.2. E2F Expression and Bevacizumab-Responsive Subtypes of Glioblastoma

To explore the potential involvement of E2Fs in the malignancy of BVZ-responsive GBM subtypes, we conducted a systematic analysis of the expression patterns of the eight E2F transcription factors and their associations with various clinicopathological features before and after BVZ treatment. Our analysis encompassed 426 GBM patients from the TCGA dataset, classified using miRNA biomarkers and a machine learning algorithm, and 17 GBM patients from the GSE79671 dataset, classified into BVZ-responsive subtypes based on CT scans conducted pre- and post-BVZ treatment.

All eight E2Fs exhibited significantly altered expression in GBM compared to normal controls, indicating their crucial association with GBM malignancy. Specifically, we observed significant upregulation of all eight E2Fs in GBM (Table 1), based on GBM and normal brain tissue specimens from the DoSurvive dataset. The log2 (FC(GBM/Normal)) values of E2F2, E2F5, E2F7, and E2F8 were 5.9, 2.37, 5.5, and 6.9, respectively.

**Figure 2:**
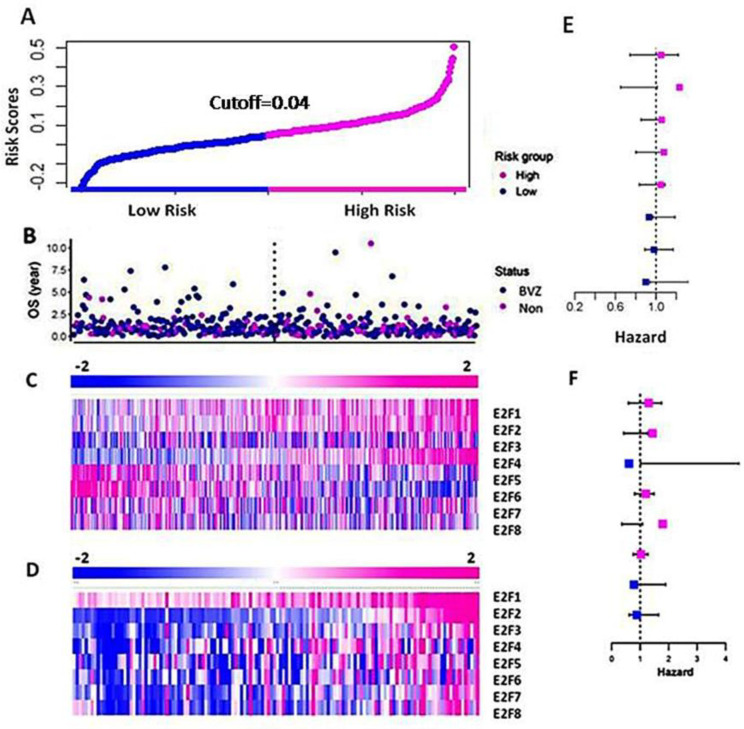
Differential expression of E2F1–8 in the high-risk and low-risk groups of GBM. (**A**) The curve of patients’ risk scores based on OS and the expression levels of E2F1–8 using the TCGA dataset. (**B**) The OS of the patients of BVZ subtypes (Blue for BVZ responsive and pink for the non-responsive subtypes of GBM). (**C**) A heatmap of expression levels of E2F1–8 from the TCGA data, grouped by their risk scores. (**D**) A heatmap of expression levels of E2F1–8 from CGGA data grouped by their risk scores. (**E**) Hazard ratios associated with E2F1–8 using TCGA data. (**F**) Hazard ratios associated with E2F1–8 using CGGA data.

Using the TAGA dataset, we constructed and performed the LASSO model and selected important members of the E2F family, namely E2F2, E2F4, and E2F8 based on their coefficients. According to Formula (1), the risk score is calculated as follows: risk score = (0.172 × E2F2 + 0.015 × E2F4 − 0.057 × E2F8). The risk score is represented in Figure 2A, with high and low risks shown in pink and blue, respectively. Figure 2B displays the patients’ OS and BVZ responsive subtypes. Additionally, Figure 2C presents a heatmap of E2F1–8 based on the risk scores. We observed no significant differences in the expression of E2F1–8 between BVZ-responsive and non-responsive GBM. In contrast, the expression levels of E2F1, E2F2, E2F4, E2F6, and 8 were significantly changed in the high-risk group compared to the low-risk group of GBM. In addition, we calculated the hazard ratios of E2F1–8 using the Cox regression method, and we found no significant difference between these eight E2F members, indicating they are equally hazardous to GBM patients, as shown in Figure 2E.

Next, we calculated and validated the risk score for the CGGA dataset using Formula (1), and we grouped the patients based on the risk score. In Figure 2D, we present a heatmap of E2F1–8 expression in the high-risk and low-risk groups consisting of 136 GBM patients. We observed a significant increase in the expression levels of E2F1–8 between the high-risk and low-risk groups. These results differ slightly from those of the TCGA dataset, as the CGGA dataset utilized a more accurate and sensitive high throughput sequencing technique, whereas the TCGA data used microarray techniques. We also calculated the hazard ratios of E2F1–8 from the CGGA data using the Cox regression method, as shown in Figure 2F. Similar to the results obtained from the TCGA data, the hazard ratios of these eight E2F members differed slightly from those of the TCGA dataset, but the difference was not significant.

### 3.3. Predicting Survival in Bevacizumab-Responsive Subtypes of Glioblastoma Using TreeBagger Analysis

Machine learning algorithms have become widely utilized for prediction and prognostic purposes in cancer research. Previously, we employed Support Vector Machine (SVM) and miRNA biomarkers to classify BVZ-responsive subtypes of GBM. However, SVM is typically suited for scenarios involving two or three variables. Our research aimed to predict the survival terms of patients with glioblastoma (GBM) who are responsive or nonresponsive to bevacizumab (BVZ) treatment using an advanced machine learning algorithm. In this study, we chose TreeBagger, a Random Forest ensemble learning method, for its ability to manage a high-dimensional dataset and to model complex, nonlinear interactions.

**Figure 3:**
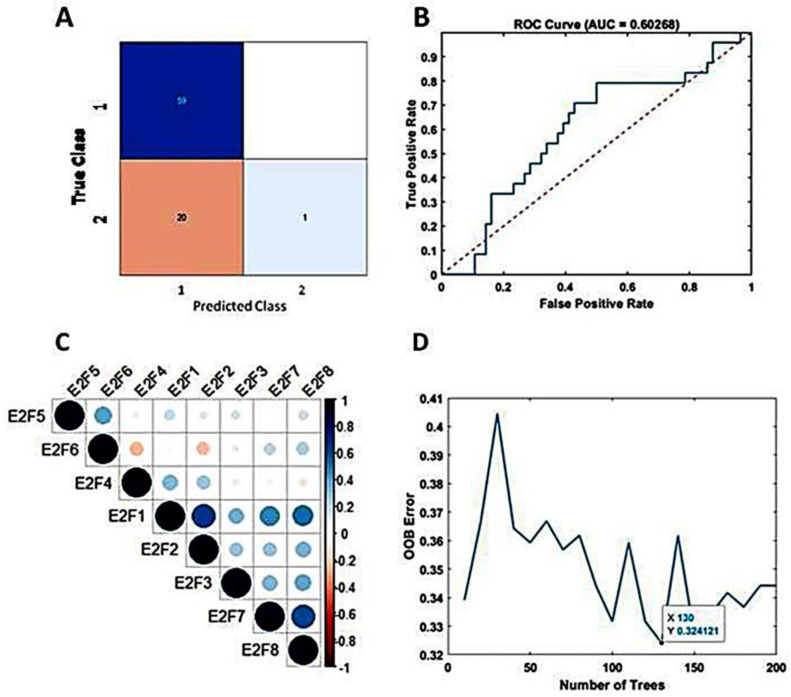
Predicting mid-term survival in BVZ-responsive glioblastoma subtypes. (**A**) Confusion matrix predicted by TreeBagger using the TCGA dataset. (**B**) Predicted ROC curve (blue). (**C**) Pearson correlation of E2F1–8 factors. (**D**)The blue line shows the optimizing process of the number of trees for the TreeBagger model.

The study incorporated all eight members of the E2F transcription factor family as predictive variables within the TreeBagger model. Despite decades of effort to optimize and combine glioblastoma treatments [38], outcomes for patients with GBM remain poor, with a median life expectancy of approximately 15 months after diagnosis [4]. In this study, we set the threshold at 15 months (450 days) to predict patient survival terms based on E2Fs expression and the machine learning analysis. Using the TCGA dataset, 80% and 20% of the dataset were set as the training set and test set, respectively. The accuracy for predicting patient survival term reached 75%. The confusion matrix illustrating the performance is presented in Figure 3A. The area under the curve (AUC) was determined to be 60%, as depicted in Figure 3B. For the calculation, the optimization of tree number (130) is shown in Figure 3D. Employing 5-fold cross-validation yielded an average accuracy of 67%, indicating no overfitting.

To validate our model, we utilized the GSE79671 dataset and applied our trained TreeBagger model to forecast survival terms before and after BVZ treatment. Prediction of patient survival before and after BVZ treatment using the GSE79671 dataset and trained TreeBagger by the TCGA dataset. When comparing the prediction of BVZ-responsive versus non-responsive GBM subtypes, this analysis did not show a significant improvement in survival after BVZ treatment, consistent with clinical observations. However, due to the small sample size, these findings necessitate further investigation.

Additionally, we explored the predictive potential of TreeBagger using a subset (E2F1, E2F2, E2F3, E2F7, and E2F8) of E2F members obtained from Pearson’s correlation analysis, as shown in Figure 3C. Specifically, we investigated the performance of TreeBagger with 150 trees and 25 leaf splits to predict patient survival using five E2F members. The results closely paralleled those obtained with all eight E2F members, with the highest accuracy reaching 73.8% and an AUC of 50%. Notably, based on the confusion matrix, E2Fs and TreeBagger algorithms showed better predictive ability in identifying survival probabilities of BVZ-responsive versus BVZ-non-responsive GBM subtypes because, as mentioned earlier [20], compared with BVZ-non-responsive patients, these patients have lower OS.

**Figure 4:**
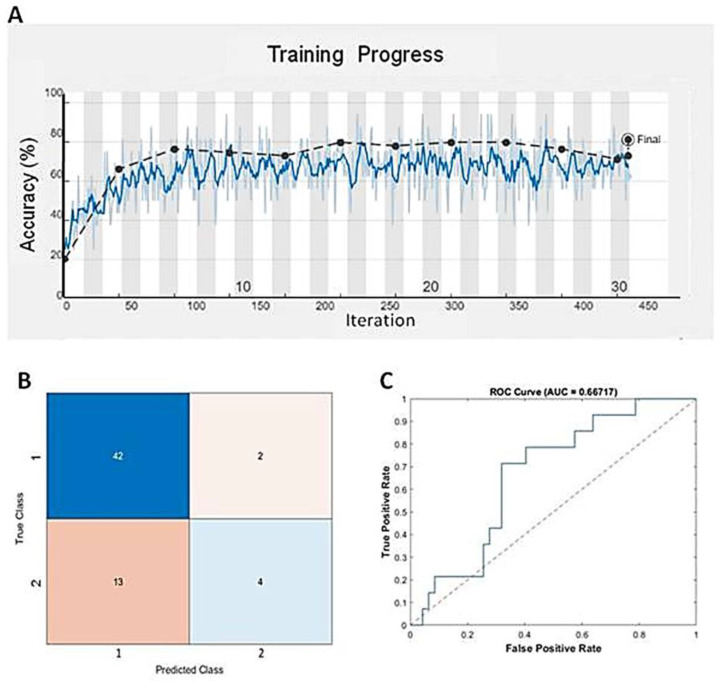
Predicting mid-term survival in glioblastoma patients using the DNN algorithm. (**A**) Training progress of the DNN model using the TCGA dataset and BVZ responsive categories. The blue line represents each calculated value, and the dashed black line represents the local average. (**B**) Confusion matrix predicted by the DNN and the dataset. (**C**) The blue line represents the predicted ROC curve using this model and dataset.

Deep learning, a specific subset of machine learning, focuses on deep artificial neural networks that can deal with complex datasets. Since we found that GBM BVZ subtypes were related to predict survival terms in TreeBagger analysis, we used the DL algorithm to combine the expression data of E2F1–8 from TCGA and BVZ responsive categories to predict the survival terms of GBM patients. After using nine input neurons, two output neurons, and 60 layers to construct a linear deep neural network (DNN), we used this DNN model to predict GBM progression. One of the training progresses of this model is shown in Figure 4A, and the validation accuracy reached to 81.4%, which is better than the results obtained by TreeBagger. The confusion matrix is shown in Figure 4B, and the ROC is shown in Figure 4C. The area under the curve (AUC) was determined to be 66.7%. Using the CGGA dataset (CGGA_mRNAseq_325) to validate this model, the accuracy reached 68.3% for 136 GBM patient survival. Considering the involvement of 106 transcription factors and 90 different TFs after BVZ treatment, the survival terms of GBM patients could be successfully predicted and validated using only E2F family members, and these findings underscore the significant role of the E2F family in tumor progression and patient survival.

### 3.4. Post-treatment Functional Analysis of E2Fs on GBM BVZ Response Subtypes

GBM frequently relapses after BVZ treatment, even in patients who respond to BVZ and experience prolongation of progression-free survival (PFS), but do not show a significant prolongation of overall survival OS [21,39]. However, there are some conflicting reports. To explore the recurrent mechanism, we performed the expression changes E2F1–8 and their related gene ontology and regulatory networks after BVZ treatment.

**Figure 5:**
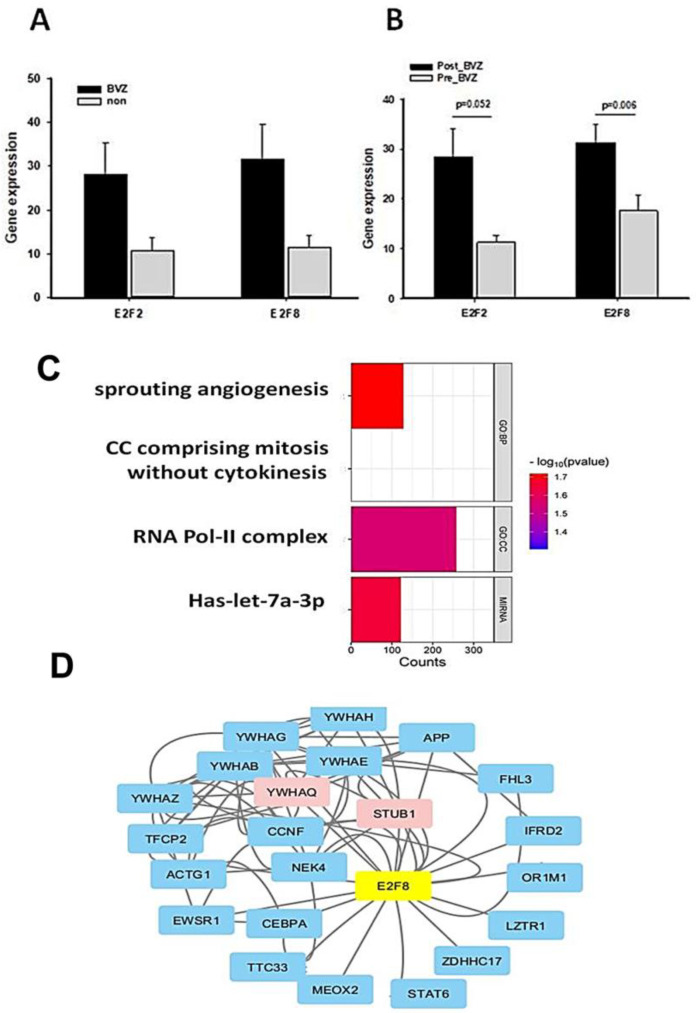
Functional and regulatory network analysis of E2F2 and E2F8 altered by BVZ treatment. (**A**) Expression levels of E2F2 and E2F8 in GBM BVZ-responsive and non-responsive subtypes before BVZ treatment from the TCGA dataset (*p* > 0.05, BVZ (n = 123) vs. Non (*n* = 286), error bars represent SE.). (**B**) Expression levels of E2F2 and E2F8 in GBM BVZ response subtype before and after treatment from GSE79671 (*p* = 0.006, *n* = 7, error bars represent SE). (**C**) Significant relevant GO terms (set at *p* = 0.01) based on E2F8 and E2F2. (**D**) Regulatory-related network of E2F8 overlapping with DE genes in BVZ responsive subtype of GBM following its treatment using Cytoscape analysis.

Similar to the TCGA dataset, there was no significant difference between BVZ-responsive and non-responsive GBM subtypes in GSE79671 before treatment, as shown in Figure 5A. However, post-BVZ treatment analysis in the GSE79671 dataset of BVZ-responsive patients showed increased expression of E2F2 and E2F8, with particular note being given to E2F8 (*p*-value = 0.006 after excluded one outlier) and E2F2 (*p*-value = 0.052), as shown in Figure 5B. In contrast, despite the presence of other DE genes, there were no significant changes in E2F2 and E2F8 before and after BVZ treatment in BVZ-non-responsive patients. These findings highlight the complex involvement of E2F family in BVZ treatment response and the underlying biological mechanisms of GBM recurrence.

We identified genes that were significantly differentially expressed before and after BVZ treatment in the BVZ-responsive subtype of GBM. We further analyzed the biological processes and cellular components associated with malignancy, as well as the genes associated with enriched miRNA in this process. In Figure 5C, the names of functional processes, gene counts, and *p*-values are shown. We observed the enrichment of genes involved in sprouting angiogenesis (GO:0002040) [40,41], cell cycle comprising mitosis without cytokinesis (GO:0033301, https://amigo.geneontology.org/amigo/term/GO:0033301/ accessed on 9 July 2024), RNA polymerase II transcription regulator complex (GO:0090575, https://amigo.geneontology.org/amigo/term/GO:0090575/ accessed on 9 July 2024), and miRNA-let-7a. In these four terms, −log(*p*-values) of sprouting angiogenesis is larger than other terms, so it was chosen for further investigation. These GO terms and pathway analyses further revealed that important genes involved in angiogenesis, cell cycle mitosis, and cell proliferation were significantly enriched in the BVZ-responsive subtype of GBM after BVZ treatment.

Furthermore, the regulatory gene network of E2F8 was explored by integrating DE gene expression in the BVZ responsive subtype of GBM before and after treatment into the current BioGrid network, as shown in Figure 5D. The protein–protein interaction (PPI) network data were organized and visualized using Cytoscape, and 25 nodes and 78 edges were found in the PPI network (all nodes are shown in Supplementary Table S2). The size and color depth of the nodes in the network correspond to their expression degree labeled by logFC values, where red indicates increased expression, green indicates decreased expression, and blue indicates no expression value in the DE genes.

The expression levels of STUB1 (STIP1 Homology And U-Box Containing Protein 1, E3 Ubiquitin) and YWHAQ (Tyrosine 3-Monooxygenase/Tryptophan 5-Monooxygenase) were significantly increased after BVZ treatment in BVZ-responsive subtype of GBM, indicating their involvement in the E2F8 network and potential regulation by E2F8. Other nodes that do not appear in the DE gene list are shown in light blue. In this figure, the colors of STUB1 and YWHAQ are shown in red, indicating that not only were their expression levels significantly increased, but they also overlapped with E2F8 regulatory networks.

### 3.5. Recurrence Machinism Analysis of E2Fs and Related Genes

**Figure 6:**
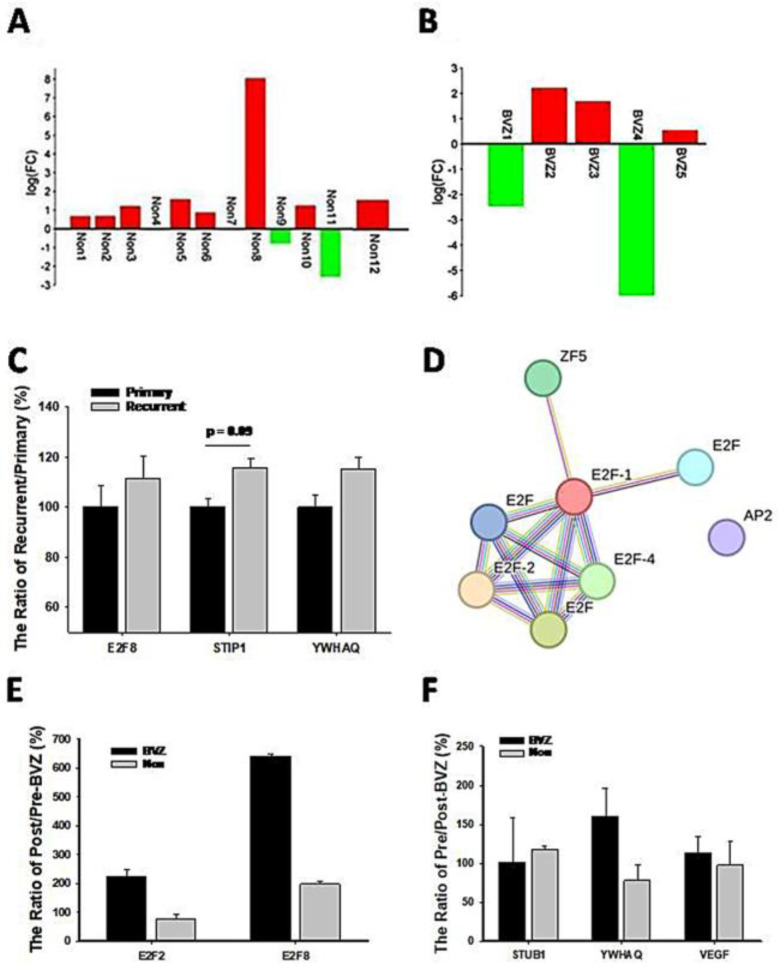
Expression levels of genes related to GBM recurrence and BVZ drug resistance. (**A**) Two-way bar graph showing the logarithm (FC) of VEGF expression after BVZ treatment in unresponsive (Non) GBM patients. (**B**) The graph shows the logarithm (FC) of VEGF expression in patients with BVZ-responsive (BVZ) GBM after BVZ treatment. (**C**) The expression levels of E2F8, STUB1, and YWHAQ from the CGGA dataset are shown as the ratio of (recurrent or primary)/primary (%) (for STUB1 *p* = 0.09, primary (82) vs. recurrent (24) and for E2F8 and YWHAQ *p* > 0.1, all error bars represent SE). (**D**) The PPI figure from STRING analysis shows that E2F members are involved as hub genes using EMBL-EBI dataset. (**E**) The expression levels of E2F2 and E2F8 represent the ratio of post- vs. pre-BVZ treatment (%) in BVZ-responsive subtypes of GBM recurrent patients after treatment (BVZ-responsive *n* = 3, BVZ-non-responsive *n* = 12, *p* > 0.05, not significant) using the EMBL-EBI dataset. (**F**) The expression levels of STUB1, YWHAQ, and VEGF represent the ratio of post- vs. pre-BVZ treatment (%) in BVZ-responsive subtypes of GBM recurrent patients after treatment (BVZ-responsive *n* = 3, BVZ-non-responsive *n* = 12, *p* > 0.05, not significant) using the EMBL-EBI dataset.

Because of the involvement of angiogenesis GO:0002040, the expression levels of VEGF in BVZ-responsive GBM subtypes after BVZ treatment were examined. There were 17 paired datasets from a total of 21 GBM samples before and after BVZ treatment, including 12 BVZ non-responsive data (Non) and 5 BVZ responsive data (BVZ). The log(FC) of (VEGFpost/VEGFpre) was calculated and presented as a bidirectional bar graph. To our surprise, 80% of patients who did not respond to BVZ treatment had increased or no change in VEGF expression after treatment, as shown in Figure 6A, while 60% of GBM patients who responded to BVZ treatment had increased VEGF expression, as shown in Figure 6B. As can be seen in the figure, there is a more than 10-fold difference in VEGF expression before and after BVZ treatment in both the subtypes that respond to BVZ and those that do not respond to BVZ, so in this sample size there is no significant difference between the two subtypes before and after BVZ treatment. However, GBM patients must be examined and considered individually. This fact suggests that BVZ treatment in unresponsive GBM patients may worsen disease progression, and accurate BVZ treatment in GBM patients is strongly recommended.

To further explore recurrent mechanisms, we compared expression levels of associated genes between recurrent and primary GBM patients. First of all, we compared the expression levels of STUB1 and YWHAQ in recurrent patients (24 cases) with primary (82 cases) GBM from the CGGA dataset, as shown in Figure 6C. Their expression levels were increased in recurrent patients, although not significantly. E2F8 expression levels also increased in comparison with primary patients, indicating their involvement in GBM recurrence. Considering that these relapsed patients did not receive BVZ treatment and were not classified into BVZ-responsive subtypes, these expression trends support our hypothesis and results to some extent, especially in the expression of STUB1. However, all these expression levels need to be validated in a larger cohort of BVZ-treated patients.

Additionally, VEGFC significantly decreased (*p* = 0.03) in recurrent patients compared with primary patients. After IDH mutant patients were excluded from both groups of GBM, E2F3 decreased significantly (*p* < 0.05) in recurrent patients, and E2F5 decreased significantly in secondary GBM IDH wild-type patients compared with primary GBM patients. In contrast, E2F8 increased in recurrent patients and secondary (10) GBM IDH wild-type patients, but not significantly in such a group size. This suggests that E2F members play different roles in GBM recurrence, though their hazard ratios are similar.

To validate these results, we used the E-MTAB-1380 and E-MEXP-3296 datasets. These datasets derived from different platforms and have a small number of cases, so the machine learning models we constructed could not be used directly in this case. Therefore, we classified and detected them based on the above-mentioned seven gene biomarkers of GBM BVZ-responsive subtypes and the original method described before [20]. We were able to use these biomarkers because they provided gene expression data from pretreatment primary GBM samples, the same conditions under which we obtained the biomarkers for GBM BVZ-responsive subtypes. Briefly, we implemented the ‘sequences’ function, one of the deep learning methods, with the TCGA dataset according to the MatLab protocol and our previous study. Then the 7 genes obtained were optimized and screened for the best variants, and GREB1 and FKBP9 were finally selected in the subsequent steps. The z scores of the highly expressed (top eight) patients for each gene before BVZ treatment were ranked. The clusters were found through the Venny diagram, giving BVZ-responsive patients as T2, T3, and T11, and the remaining patients as BVZ-non-responsive subtypes of GBM. Supplementary Figure S2 shows the selection process. The Venn diagram showed that patient IDs among patients with high expression of both GREB1 and FKBP9 were clustered as BVZ-responsive GBM patients. The other patient IDs (highly expressed GREB1 or highly expressed FKBP9) were BVZ-non-responsive patients. Considering that these patients who received BVZ treatment relapsed after an average of 31 days, it is reasonable that 20% of patients in this group responded to BVZ treatment [33], even though the proportion was approximately 30%, based on previous AI and clinical studies [20,31,42].

After categorizing the 15 GBM patients into BVZ-responsive and non-responsive subtypes, we identified 1249 differentially expressed (DE) genes in the responsive subtype (*n* = 3) and 1120 DE genes in the non-responsive subtype (*n* = 12), using paired *t*-tests with a significance level of *p* < 0.05. Functional analysis of these DE genes revealed eight transcription factors (TFs) that surpassed the significance threshold in the BVZ-responsive subtype. In contrast, as shown previously, again no significant TFs were identified in the non-responsive subtype. We further analyzed these significant TFs using STRING analysis, as shown in Figure 6D. Despite identifying only eight TFs, including members of the E2F family such as E2F1, E2F2, E2F4, and E2F family, these E2F members still emerged as hub genes in the regulatory network. It is noteworthy that the E2F family appeared twice in the list as before, representing different TF IDs and associated with distinct targets. This reflects the complexity of TF regulation, which is intricately linked to sequence senses. It is important to acknowledge the significant experimental differences between the GSE79671 and EMBL-EBI datasets. These differences include high-throughput RNA-seq technology versus microarray technology, fresh tissue versus PPFE fixed tissue, and seven cases versus three cases. Most importantly, these patients were used for BVZ resistance studies, and their tumors relapsed within an average of 31 days. Therefore, the validation of the E2F family regulatory network should be considered to have been well reproduced using this dataset. Furthermore, the results again demonstrate that the E2F family regulatory network is not only important but also sensitive to different situations.

To further explore the mechanism of recurrence, we compared the expression levels of relevant genes in GBM patients before and after BVZ treatment in the EMBL-EMI dataset. First, we compared the expression levels of E2F2 and E2F8 in the BVZ-responsive subtypes of recurrent patients, as shown in Figure 6E. In BVZ-responsive GBM patients, the expression levels of E2F2 and E2F8 increased by approximately 2-fold and 6.5-fold, respectively, after BVZ treatment. In contrast, in BVZ-unresponsive patients, the expression level of E2F2 decreased by 70% compared to that before BVZ treatment, while the expression levels of E2F8 increased by approximately 190%. Then, we examined the expression levels of STUB1, YWHAQ, and VEGF in BVZ-responsive subtypes of GBM recurrent patients, as shown in Figure 6F. The expression levels of YWHAQ increased by 160% in BVZ-responsive GBM recurrent patients but decreased by 70% in BVZ-non-responsive GBM recurrent patients after BVZ treatment. The expression levels of STUB1 and VEGF remained unchanged or slightly increased in BVZ-responsive subtypes of GBM recurrent patients. Although there were many differences between this dataset and the previously used dataset, these correlated gene expression data partially supported our previous results, especially in the expression of E2F2, E2F8, and YWHAQ. After treatment, the expression levels of E2F8 and E2F2 increased by 190% and 118, respectively, and the expression levels of STUB1 and YWHAQ increased by approximately 110% between GBM BVZ-responsive and non-responsive subtypes (*p* > 0.05, BVZ (*n* = 3) vs. Non (*n* = 12)). These results suggest that the expression levels of these related genes were increased in patients with relapse after BVZ treatment but were more increased in GBM BVZ-responsive patients. Therefore, all results were repeated and validated to some extent, including the regulatory networks of the E2F family, the expression levels of E2F2, E2F8, STUB1, and YWHAQ after BVZ treatment.

## 4. Discussion

This study introduced a novel perspective by focusing on the complex relationship between E2F family members, survival outcomes, and relapse mechanisms in GBM patients after BVZ treatment, with a particular emphasis on the role of E2F8. BVZ treatment has been shown to have a significant impact on 90 transcription factors and their hub genes, including E2Fs. This phenotype encouraged our investigation. The expression levels of all E2F members have a strong association with survival-related risk, but the more important factors identified by the LASSO model were E2F8, E2F2, and E2F4. Using machine learning approaches, including TreeBagger and DNN, and E2F1–8 expression datasets, survival terms of GBM patients were successfully predicted and validated, emphasizing that these factors play a key role in tumor progression and patient survival. By analyzing clinicopathological characteristics in primary and recurrent GBM, we gained insights and identified targets related to angiogenesis and proliferation through E2F8 regulation after BVZ treatment. Most importantly, BVZ therapy may worsen disease progression in patients with unresponsive GBM, indicating the necessity of precise treatment. Finally, we were the first to identify and validate the involvement of STUB1 and YWHAQ, suggesting their potential roles in GBM recurrence and progression in patients who are responsive to BVZ treatment. These findings may guide future therapeutic strategies. However, further experimentation is needed to confirm these results derived from bioinformatics and artificial intelligence analyses.

Given that AI technology has been successfully used in precision medicine, including in cancer category classification and drug design based on big data training and validation [43,44], this study utilized AI technology, bioinformatics analyses, and existing datasets as tools and resources. It followed the principles of real-world experiments (or wet experiments), which involve starting with new phenotypes and open exploration to explore possible GBM recurrence mechanisms. Throughout this process, researchers constantly tracked new data and clues, adjusted directions, and improved their understanding. The only difference here is the use of artificial intelligence algorithms and existing datasets as tools. Most importantly, AI techniques and bioinformatics analyses often deliver surprising results and opportunities because they avoid barriers such as funding, equipment and spaces, and biases stemming from personal preferences or perspectives. This novel journey has brought existing research, accumulated through years of human effort and vast amounts of money, back to life, contributing to human well-being and biomedical exploration again. In contrast, real experiments emphasize repeatability and control. Our findings have been validated and compared using different datasets from various research groups, thus demonstrating repeatability and reproduction of our results through a combined validation approach. This study not only discovered novel facts and possible mechanisms, but also contributed to customizing and adapting existing interpretable ML methods and datasets to biomedical and bioinformatics research problems. It provided suggestions and directions for precision cancer treatment and research.

Previous studies have indicated that E2F7 and E2F8, both considered as atypical E2Fs, function as transcriptional regulators of VEGF expression [45]. Specifically, they directly bind to, and stimulate, the VEGF promoter, independently of canonical E2F binding elements, thereby controlling angiogenesis. BVZ, an antibody that neutralizes and inhibits VEGF, thus suppressing angiogenesis, has been found to potentially increase E2F8 expression. This increase in E2F8 expression may reduce the effectiveness of BVZ treatment, rapidly restore VEGF expression and angiogenesis, and increase its hazard of risk. In recurrent GBM, E2F8 also showed a tendency to increase, while E2F3 decreased significantly. However, the underlying mechanisms responsible for BVZ enhancing E2F8 expression remain unknown. Furthermore, studies on mouse and zebrafish with non-functional E2F7/8 mutant have demonstrated varying degrees of defects in angiogenesis and apoptosis, highlighting the crucial role of E2F7/8 in angiogenesis. In a mouse model, the inhibition of E2F8 has been shown to suppress the development of various tumors by inhibiting angiogenesis [46]. Therefore, incorporating E2F8 inhibition into BVZ therapy may potentially enhance the inhibition of angiogenesis and lead to improved treatment efficiency.

E2F8 plays a significant role in cancer by affecting the proliferation and differentiation of cancer stem cells (CSCs). CSCs are a small group of cancer cells that can self-renew and give rise to a diverse lineage of tumor-forming cells. They are largely responsible for tumor growth, progression, and recurrence [47]. Studies have shown that E2F8 is upregulated in various cancers, including glioblastoma, ovarian cancer, and hepatocellular carcinoma, where it promotes cell proliferation and other effects [15,48]. Moreover, the regulation of E2F8 throughout the cell cycle demonstrates extensive co-ordination between phosphorylation, ubiquitination, and transcription in the mammalian cell cycle. E2F8 is involved in the regulation of cyclin D1 and promotes the accumulation of S-phase cells in hepatocellular carcinoma, further highlighting its role in cell proliferation [49]. Additionally, E2F8 is one of the E2F family of transcription factors and is crucial for various cellular processes, including those associated with cancer stem cells [8]. Therefore, targeting E2F8 may present a promising therapeutic approach to disrupt cancer stem cells and hinder tumor growth and progression.

Furthermore, STUB1, an E3 ligase gene, is encoded at p13.3 of chromosome 16. It contains seven exons and six introns and encodes a protein consisting of 303 amino acids [50]. It plays a crucial role in cancer by regulating multiple pathways and participating in the E2F8 regulatory network. In GBM-recurrent patients, STUB1 expression increased. After BVZ treatment, it increased significantly, indicating its effects in recurrent GBM and as resistant to BVZ treatment. This study revealed that STUB1 inhibitors suppress interferon gamma (IFNγ) responses by degrading IFNγ receptor 1 (IFNGR1). As a result, STUB1 inhibitors enhance the tumor response to checkpoint immunotherapy [51]. Additional studies have demonstrated that knocking down STUB1 increases the likelihood of metastasis to the lungs of mice when injected intravenously or subcutaneously. In breast and lung cancer, STUB1 interacts with various proteins through its ubiquitination-dependent proteasome activity. It acts as a negative regulator associated with different proteins, affecting cell cycle progression and metastasis [52,53].

YWHAQ belongs to the 14-3-3 (YWHA) small protein family with a molecular weight of approximately 30 kD and has a hockey stick shape. The gene is located at 8q22.3. Various 14-3-3 isoforms have highly conserved structures and significant sequence similarity, so they were historically studied using a single isoform [54]. They are phospho-serine/-threonine binding proteins that are highly conserved and participate in many important cellular processes, including metabolism, protein transport, signal transduction, apoptosis, and cell cycle regulation. After BVZ treatment, YWHAQ expression levels were significantly increased, and its expression was also increased in recurrent GBM patients. It may play an important role in GBM recurrence. In addition, YWHAQ is upregulated in patients with amyotrophic lateral sclerosis (ALS) [55]. While its specific expression in cancer has not been extensively reported, other members of the family have been studied in cancer. For example, YWHAZ, also known as 14-3-3ζ, is frequently upregulated in various cancers and acts as an oncogene, promoting tumor progression through multiple cellular activities. In breast cancer tissues, the expression levels of DAAM1 (dishevelled-associated activator of morphogenesis 1) and YWHAZ are significantly upregulated and colocalized, with YWHAZ binding to DAAM at its phosphorylated site, regulating cancer cell migration and being strongly associated with poor prognosis [56]. The WHO now recognizes four categories of endometrial stromal tumor, with YWHAE, another 14-3-3 protein translocation, identifying high-grade endometrial stromal sarcoma (HG-ESSs). This highlights the impact of the YWHA family in molecular genetics in cancer and supports a new classification system [57].

Based on numerous works of research, including our studies, accurate BVZ treatment is highly recommended, for there are only about 30% of GBM patients who respond to it [20,31,42]. For BVZ-non-responsive patients, they will take serious risks but without benefits. Our previous study showed that BVZ treatment in unresponsive patients of GBM resulted in multiple side effects, including aging [26]. In this study, we found that BVZ treatment is most likely to increase VEGF expression, particularly in patients with BVZ-unresponsive GBM. This VEGF increase may cause tumor recurrence and BVZ resistance. Furthermore, according to clinical reports, GBM patients who undergo BVZ treatment are at risk of wound complications, including dehiscence, cerebrospinal fluid leakage, infection, etc. After treatment, 44% of GBM patients who received pre-operative BVZ developed severe healing complications, compared to only 9% of patients who did not receive treatment, which significantly increased the morbidity and mortality of these patients [58]. Other serious adverse events that GBM patients receiving BVZ treatment reported included hypertension, neutropenia, visceral perforation, and serious hemorrhage [59]. This fact is also supported by some reports about other cancers. It was reported that VEGF expression significantly increased in SKBR-3 breast cancer cells treated with BVZ compared with untreated cells, both in the low- and high-estrogen groups [60]. Moreover, BVZ treatment increased VEGF in endothelial cells isolated from colon cancer [61]. Additionally, VEGF levels were found to be elevated in the blood of patients with colorectal cancer metastases treated with BVZ, which was thought to be due to a blockade of VEGF clearance by the host [62]. Therefore, this increase may lead to GBM recurrence and affect the OS of GBM patients treated with BVZ. In patients who responded to BVZ, the treatment decreased several angiogenesis-related genes in addition to VEGF, including Ang1, SH1D2A, and LEF1, as we previously reported, although VEGF expression was increased in some patients, which may indicate some different mechanisms, such as VEGF as an independent mechanism for its antiangiogenic effects.

The utilization of AI techniques and pre-existing datasets in bioinformatics and biomedical research studies is not without challenges [63], despite their discussed advantages. The quality of AI’s output is inherently tied to the data it is trained on, which may contain biases or inaccuracies, especially biomedical data. The training process also leads to a bias-variance trade-off, which is common to all machine learning applications. Reliance on existing data can also hinder the discovery of new findings because extensively studied datasets may not yield groundbreaking insights. In addition, some data are generated using different techniques and are small in size, making them difficult to combine and validate against each other, as we encountered in this study. Furthermore, some machine learning models, such as deep neural networks, are black boxes, making it difficult to interpret their decision-making processes. The lack of experimental validation raises questions about the biological relevance of computational predictions. Additionally, the use of pre-packaged software, such as some of those listed in the Materials and Methods section (without coding), can limit customization and perpetuate existing biases within the tools. Additionally, there are legal and privacy restrictions when dealing with sensitive health records and exposure risks. The rapid evolution of AI technology can render research quickly obsolete, while the resource-intensive nature of AI models can be a barrier for some researchers. Lastly, the statistical significance identified by AI does not always indicate biological relevance, leading to misleading conclusions. Addressing these limitations requires a conscientious approach to research design, transparency and, whenever possible, experimental corroboration of computational results.

## 5. Conclusions

In conclusion, a deep examination of E2F members provides new insights into the mechanisms of glioblastoma outcomes and recurrence after bevacizumab (BVZ) treatment, specifically those associated with E2F8. This study provides very strong evidence to recommend precision BVZ treatment for GBM patients. In addition, the involvement of STUB1 and YWHAQ was revealed for the first time. However, there is still much to be explored and confirmed in the future, especially regarding the changes in TFs after BVZ treatment. It is worth noting that, although the E2F family positively influences angiogenesis, proliferation, and cancer progression, it also regulates some negative regulators or tumor suppressors, and each member of the family shows different sensitivities to different pathological situations. This leads to complex regulation of tumor growth and diverse responses to cancer treatments. Therefore, targeting hub genes like E2F8 or multiple targets simultaneously may yield favorable outcomes in cancer treatment.

## Figures and Tables

**Table 1:** E2F1–8 Expression in Glioblastoma.

Gene	Normal	GBM	GBM/Normal	Log(FC)	Wilcoxon *p*-Value
E2F1	2.42 × 10^+02^	6.02 × 10^+02^	2.49 × 10^+00^	1.31 × 10^+00^	9.99 × 10^−03^
E2F2	3.14 × 10^+00^	1.92 × 10^+02^	6.11 × 10^+01^	5.93 × 10^+00^	1.43 × 10^−04^
E2F3	4.65 × 10^+02^	5.96 × 10^+02^	1.28 × 10^+00^	3.58 × 10^−01^	4.76 × 10^−01^
E2F4	7.18 × 10^+02^	1.05 × 10^+03^	1.46 × 10^+00^	5.48 × 10^−01^	1.50 × 10^−03^
E2F5	8.48 × 10^+01^	4.39 × 10^+02^	5.18 × 10^+00^	2.37 × 10^+00^	1.43 × 10^−04^
E2F6	3.07 × 10^+02^	5.56 × 10^+02^	1.81 × 10^+00^	8.57 × 10^−01^	3.27 × 10^−04^
E2F7	4.37 × 10^+00^	1.91 × 10^+02^	4.37 × 10^+01^	5.45× 10^+00^	1.55 × 10^−04^
E2F8	6.50 × 10^−01^	7.65 × 10^+01^	1.18 × 10^+02^	6.88× 10^+00^	1.55 × 10^−04^

## Data Availability

The resource and links of original data have been provided in the Materials and Methods section, including their inclusion and exclusion criteria.

## Supplementary Materials

The following supporting information can be downloaded at: https://www.mdpi.com/article/10.3390/cancers16142536/s1, Figure S1: Differential Networks between TFs; Figure S2: The selection of BVZ responsive patients from E-MTAB-1380; Table S1: The list of 106 and 90 TFs between BVZ-pre and -post; Table S2: E2F8 network nodes.
