# Supplementary material for: Early 2-Factor Transcription Factors Associated with Progression and Recurrence in Bevacizumab-Responsive Subtypes of Glioblastoma
Source: Cancers (Basel). 2024 Jul 14;16(14):2536. doi: 10.3390/cancers16142536 (PMC11275000; doi:10.3390/cancers16142536)
Supplement: Supplementary file 1 [file cancers-16-02536-s001.zip › Supplementary Table S2 E2F8 network nodes.pdf]

Supplementary Table S2 E2F8 network nodes

SUID	adj-pvalue	FDR	Interactor Type	log(FC)	name	selected	shared name	synonym
8563			protein		CCNF	FALSE	CCNF	FBXO1
2355			protein		MEOX2	FALSE	MEOX2	MOX2
5944	0.0135	-238.858	protein	0.811733	STUB1	FALSE	STUB1	UBOX1
10173			protein		LZTR1	FALSE	LZTR1	SWNTS2
1469			protein		EWSR1	FALSE	EWSR1	bK984G1.4
9022			protein		IFRD2	FALSE	IFRD2	SM15
1535			protein		YWHAG	FALSE	YWHAG	PPP1R170
40321			protein		OR1M1	FALSE	OR1M1	OR19-6
36545			ribonucleic acid		E2F8	FALSE	E2F8	E2F-8
259			protein		ZDHHC17	FALSE	ZDHHC17	HYPH
5380			protein		STAT6	FALSE	STAT6	STAT6C
133			protein		YWHAZ	FALSE	YWHAZ	YWHAD
2950			protein		YWHAB	FALSE	YWHAB	YWHAA
262	0.026	-229.622	protein	0.759781	YWHAQ	FALSE	YWHAQ	HS1
1607			protein		FHL3	FALSE	FHL3	SLIM2
266			protein		YWHAE	FALSE	YWHAE	MDS
3917			protein		CEBPA	FALSE	CEBPA	CEBP
79			protein		APP	FALSE	APP	PN2
10200			protein		E2F8	TRUE	E2F8	E2F-8
11481			protein		TTC33	FALSE	TTC33	OSRF
18524			protein		NEK4	FALSE	NEK4	pp12301
541			protein		NONO	FALSE	NONO	PPP1R114
477			protein		TFCP2	FALSE	TFCP2	TFCP2C
4836			protein		YWHAH	FALSE	YWHAH	YWHA1
1770			protein		ACTG1	FALSE	ACTG1	HEL-176
