# Supplementary material for: Early 2-Factor Transcription Factors Associated with Progression and Recurrence in Bevacizumab-Responsive Subtypes of Glioblastoma
Source: Cancers (Basel). 2024 Jul 14;16(14):2536. doi: 10.3390/cancers16142536 (PMC11275000; doi:10.3390/cancers16142536)
Supplement: Supplementary file 1 [file cancers-16-02536-s001.zip › SupplementaryTable S1-BVZpreVSpostTF106.pdf]

source	term_name	term_id	adj-p	neg-log-adj-p	term_size	inters_size
TF	Factor: Churchill; motif: CGGGNN	TF:M0098	1.68E-10	9.774817466	10709	328
TF	Factor: ZXDL; motif: GSGSCNNGG	TF:M1272	2.94E-10	9.532128939	10881	331
TF	Factor: E2F-2; motif: GCGCGCGCN	TF:M1152	2.58E-09	8.587544115	14894	409
TF	Factor: ETF; motif: GVGGMGG	TF:M0069	2.63E-09	8.580765309	10745	325
TF	Factor: ETF; motif: GVGGMGG; m	TF:M0069	1.43E-08	7.843634421	7249	241
TF	Factor: E2F4; motif: YCCCGCCNCN	TF:M1259	4.22E-08	7.374989025	16076	427
TF	Factor: ZF5; motif: NRNGNGCGCG	TF:M0033	1.18E-07	6.927107256	12784	363
TF	Factor: E2F-2; motif: GCGCGCGCN	TF:M1152	1.21E-07	6.918658449	16674	436
TF	Factor: Egr-1; motif: GCGCATGCG	TF:M0486	1.36E-07	6.866482124	10557	315
TF	Factor: ZF5; motif: GGSGCGCGS; r	TF:M1043	2.24E-07	6.649883654	15279	411
TF	Factor: ZF5; motif: NRNGNGCGCG	TF:M0033	3.12E-07	6.506084	15464	414
TF	Factor: E2F-2; motif: GCGCGCGCG	TF:M1153	4.24E-07	6.373013847	13659	379
TF	Factor: RNF96; motif: BCCCGCRG(	TF:M0119	4.94E-07	6.305985633	4481	164
TF	Factor: GCMa:Erg; motif: ATGCGG	TF:M0848	7.27E-07	6.138249386	14303	391
TF	Factor: WT1; motif: GNGGGGGCG	TF:M0389	7.45E-07	6.128036341	8544	266
TF	Factor: Egr-1; motif: GCGCATGCG	TF:M0486	1.07E-06	5.972625091	11569	334
TF	Factor: E2F-2; motif: GCGCGCGCG	TF:M1153	1.23E-06	5.908851754	12708	358
TF	Factor: RREB-1; motif: GGGWCSA	TF:M0543	1.58E-06	5.800842118	8852	272
TF	Factor: ZF5; motif: GSGCGCGR; m	TF:M0071	1.79E-06	5.74765359	14519	394
TF	Factor: MOVO-B; motif: GNGGGG	TF:M0110	2.01E-06	5.697371589	10571	311
TF	Factor: Churchill; motif: CGGGNN	TF:M0098	2.02E-06	5.694577726	14579	395
TF	Factor: TCF-1; motif: ACATCGRGR	TF:M1160	2.57E-06	5.590301383	11404	329
TF	Factor: WT1; motif: CGCCCCCNCN	TF:M0203	2.61E-06	5.583149942	10058	299
TF	Factor: ZF5; motif: GGSGCGCGS	TF:M1043	2.79E-06	5.553783486	16621	432
TF	Factor: E2F-4; motif: SNGGGCGG(	TF:M0989	5.68E-06	5.245688451	14345	389
TF	Factor: E2F-3; motif: GGCGGGN	TF:M0208	6.69E-06	5.174860201	13559	373
TF	Factor: RNF96; motif: BCCCGCRG(	TF:M0119	6.95E-06	5.157917608	8748	267
TF	Factor: ETF; motif: CCCCGCCCCYN	TF:M0703	1.09E-05	4.962896211	16764	433
TF	Factor: E2F-1:HES-7; motif: GGCR	TF:M0852	1.44E-05	4.840633605	15663	413
TF	Factor: E2F4; motif: YCCCGCCNCN	TF:M1259	1.45E-05	4.838601299	17574	446
TF	Factor: ZNF37A; motif: CCYYGGCT	TF:M1235	2.17E-05	4.66434282	11203	321
TF	Factor: ZF5; motif: GSGCGCGR	TF:M0071	3.29E-05	4.483168432	16490	427
TF	Factor: E2F-4; motif: SNGGGCGG(	TF:M0989	3.41E-05	4.466740031	16606	429
TF	Factor: TCF-1; motif: ACATCGRGR	TF:M1160	3.81E-05	4.419148134	13544	370
TF	Factor: PATZ; motif: GGGGNGGG(	TF:M1002	3.96E-05	4.402511624	8669	262
TF	Factor: E2F; motif: GGCGSG	TF:M0080	5.06E-05	4.296157384	13566	370
TF	Factor: BTEB3; motif: CCNNSCCNS	TF:M0982	6.37E-05	4.196170745	11837	333
TF	Factor: AP2; motif: GCCYGSGGSN	TF:M0886	7.12E-05	4.147535953	10315	299
TF	Factor: E2F-1:HES-7; motif: GGCR	TF:M0852	7.29E-05	4.137493878	17481	443
TF	Factor: ETF; motif: CCCCGCCCCYN	TF:M0703	8.09E-05	4.092036193	13994	378
TF	Factor: AP-2alpha; motif: NGCCYS	TF:M0185	0.000111	3.955801224	9435	278
TF	Factor: TCF-1; motif: ACATCGRGR	TF:M1160	0.000113	3.947250056	15178	401
TF	Factor: EGR1; motif: NCNCCGCCC	TF:M0720	0.000133	3.876394187	6380	204
TF	Factor: SP1:SP3; motif: CCSCCCCC	TF:M0121	0.000138	3.861150986	7026	220
TF	Factor: TIEG1; motif: NCCCNSNCC	TF:M1235	0.000147	3.831737569	12696	350
TF	Factor: E2F-3:HES-7; motif: NNNS	TF:M0852	0.000155	3.811061923	17226	438

TF	Factor: E2F-1; motif: NGGGCGGG	TF:M0720	0.000159	3.799086335	11184	317
TF	Factor: MOVO-B; motif: GNGGGG	TF:M0110	0.000167	3.778153449	5843	190
TF	Factor: KROX; motif: CCCGCCCCC	TF:M0098	0.000175	3.757534752	8073	245
TF	Factor: E2F-1; motif: WWTGGCG	TF:M0451	0.000194	3.713295241	12531	346
TF	Factor: GCMa:Erg; motif: ATGCG	TF:M0848	0.000204	3.691271393	9056	268
TF	Factor: E2F3; motif: NNRGMKGG	TF:M1259	0.000244	3.612314432	7152	222
TF	Factor: ZNF138; motif: GCAGCRS	TF:M1314	0.00027	3.568166758	9856	286
TF	Factor: MAZ; motif: GGGMGGGG	TF:M0963	0.000299	3.524657331	14493	386
TF	Factor: E2F1; motif: GSGCGGGAA	TF:M1259	0.000353	3.452004024	14407	384
TF	Factor: E2F-3:TBR2; motif: ANGT	TF:M0820	0.000395	3.403063277	15846	412
TF	Factor: ZXDL; motif: GSGSCNNGG	TF:M1272	0.000408	3.388986566	6868	214
TF	Factor: E2F-1; motif: NGGGCGGG	TF:M0720	0.000416	3.381414627	14971	395
TF	Factor: ZNF253; motif: SNGNSCG	TF:M1314	0.000426	3.370676263	11358	319
TF	Factor: E2F-3:HES-7; motif: NNNS	TF:M0852	0.00045	3.347010856	15386	403
TF	Factor: Sp2; motif: TGGGCGCGCC	TF:M0694	0.000457	3.340066598	9124	268
TF	Factor: Sp1; motif: GGGGCGGGG	TF:M0093	0.000464	3.33376506	10605	302
TF	Factor: E2F-2; motif: NWTTTGGC	TF:M1153	0.000466	3.331739422	14980	395
TF	Factor: E2F-1; motif: WWTGGCG	TF:M0451	0.000518	3.285633785	13705	369
TF	Factor: E2F; motif: GGCGSG; mat	TF:M0080	0.000574	3.241180406	10535	300
TF	Factor: HA95; motif: CCSNSSCCNS	TF:M1312	0.000607	3.216714397	12581	345
TF	Factor: KLF15; motif: RCCMCRCC	TF:M1216	0.000681	3.166620836	13060	355
TF	Factor: Egr-1; motif: GCGGGGGC	TF:M0735	0.000735	3.133852388	8150	244
TF	Factor: Egr-1; motif: GCGGGGGC	TF:M0187	0.000926	3.03362356	6934	214
TF	Factor: EZI; motif: GGGGAGGGGR	TF:M1015	0.000934	3.029440577	2201	88
TF	Factor: Miz-1; motif: NNRGGWG	TF:M1011	0.000958	3.018581912	8762	258
TF	Factor: E2F-1; motif: NNNNGGCG	TF:M0989	0.001136	2.944633632	13916	372
TF	Factor: AP-2alpha; motif: NGCCYS	TF:M0185	0.001355	2.868204903	4757	158
TF	Factor: GKLF; motif: NNNRGGNG	TF:M0728	0.001439	2.841872608	15020	394
TF	Factor: Sp1; motif: NNGGGGCGG	TF:M0093	0.00145	2.838727951	10750	303
TF	Factor: ZIC4; motif: NNCCNCCCRY	TF:M1222	0.001455	2.837281507	11376	317
TF	Factor: E2F-1:Elk-1; motif: SGCGC	TF:M0820	0.001517	2.819067818	14824	390
TF	Factor: ZNF670; motif: SNGGGCR	TF:M1312	0.001675	2.775991436	13091	354
TF	Factor: GKLF; motif: NNRRGRRNG	TF:M0704	0.002075	2.682910532	13016	352
TF	Factor: KLF15; motif: NCCMCGCC	TF:M1215	0.002285	2.641044402	11372	316
TF	Factor: Sp1; motif: NGGGGGCGG	TF:M0019	0.002308	2.636834406	10571	298
TF	Factor: Sp1; motif: CCCCGCCCCN	TF:M0093	0.002413	2.617434938	10005	285
TF	Factor: AP-2gamma; motif: GCCY	TF:M0047	0.002473	2.606825779	9660	277
TF	Factor: MAZ; motif: GGGMGGGG	TF:M0963	0.002825	2.548973565	16635	424
TF	Factor: E2F-1; motif: NNNNGGCG	TF:M0989	0.002935	2.532374785	9460	272
TF	Factor: E2F-2; motif: NWTTTGGC	TF:M1153	0.002984	2.525175156	13807	368
TF	Factor: MAZ; motif: GGGGGAGG	TF:M0998	0.003201	2.494673432	5755	182
TF	Factor: Miz-1; motif: NNRGGWG	TF:M1011	0.003268	2.485665025	4064	138
TF	Factor: CPBP; motif: GNNRGGGH	TF:M0997	0.003349	2.475043325	11183	311
TF	Factor: BEN; motif: CAGCGRNV	TF:M0124	0.00353	2.452236916	16870	428
TF	Factor: BEN; motif: CAGCGRNV; m	TF:M0124	0.003644	2.438397533	13586	363
TF	Factor: ZNF219; motif: SNNCAGC	TF:M1314	0.004027	2.394979368	11695	322
TF	Factor: EGR; motif: CGCCCCCGCN	TF:M0887	0.004416	2.354937817	7724	230

TF	Factor: WT1; motif: RGGNGGGGG	TF:M1010!	0.00479	2.319681461	2954	107
TF	Factor: TR4; motif: ACCCCGS; mat	TF:M0493	0.005337	2.272716241	15788	407
TF	Factor: TCF-1; motif: ACATCGRGR	TF:M1160	0.005354	2.271286455	16683	424
TF	Factor: WT1; motif: RGGNGGGGG	TF:M1010!	0.005776	2.238361075	6724	205
TF	Factor: p300; motif: ACNTCCG; m	TF:M0482	0.00693	2.159274532	15605	403
TF	Factor: BTEB3; motif: CCNNSCCNS	TF:M0982	0.007257	2.139260464	7769	230
TF	Factor: WT1; motif: SMCNCCNSC	TF:M0111	0.007372	2.132413348	7030	212
TF	Factor: Osx; motif: CCNCCCCCNNI	TF:M0732	0.008619	2.064547626	7044	212
TF	Factor: Sp6; motif: WGGGCGG	TF:M0536	0.008751	2.057965056	8793	254
TF	Factor: Sp2; motif: WGGGCGG	TF:M0533	0.008751	2.057965056	8793	254
TF	Factor: CPBP; motif: NGGGCGG	TF:M0544	0.008751	2.057965056	8793	254
TF	Factor: GKLF; motif: NNNRGGNGI	TF:M0728	0.008997	2.045925376	10876	302
TF	Factor: E2F-1:Elk-1; motif: SGCGC	TF:M0820	0.009067	2.042524047	10744	299
