# Supplementary figures and images for: Early 2-Factor Transcription Factors Associated with Progression and Recurrence in Bevacizumab-Responsive Subtypes of Glioblastoma
Source: Cancers (Basel). 2024 Jul 14;16(14):2536. doi: 10.3390/cancers16142536 (PMC11275000; doi:10.3390/cancers16142536)

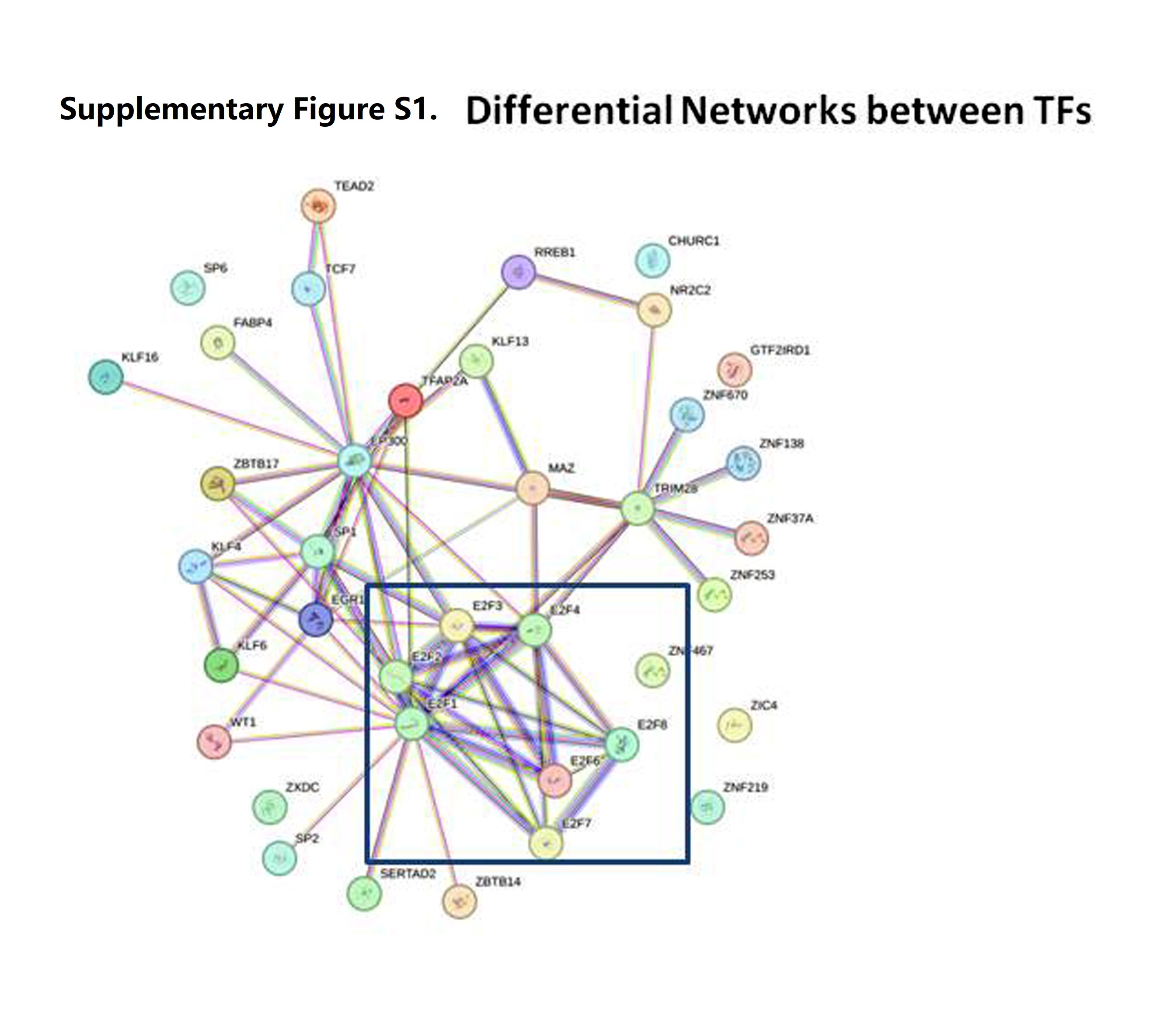

Supplement: Supplementary file 1 [file cancers-16-02536-s001.zip › SupplementaryFigure S1.jpg]
